# Therapeutic Response of Alopecia Areata-Associated Nail Changes to Baricitinib

**DOI:** 10.1155/2024/8879884

**Published:** 2024-08-31

**Authors:** Ashley Wittmer, Katherine De Jong, Lauren Bolish, Lindsey Finklea

**Affiliations:** ^1^ Texas A&M University School of Medicine, Bryan, TX, USA; ^2^ Trinity University, San Antonio, TX, USA; ^3^ University of Texas Health Science Center at San Antonio, San Antonio, TX, USA

## Abstract

Nail changes are seen in some individuals with alopecia areata, with the most common variants including pitting and trachyonychia. The nail findings are presumed to be due to the same lymphocytic infiltration seen in hair bulbs in individuals with AA. Baricitinib is an immunomodulatory drug that acts as a selective and reversible inhibitor of JAK proteins and is indicated for adult patients with moderate to severe rheumatoid arthritis who have not responded to other disease-modifying antirheumatic drugs. The FDA has also approved baricitinib to treat patients hospitalized with COVID-19 and severe alopecia areata. In this report, we present a case of a patient with persistent AA-associated nail changes who has been successfully treated with baricitinib. The patient has been suffering from alopecia for several years. She presented with periungual inflammation in conjunction with persistent fingernail ridges and pitting of her right fourth digit. The nail dystrophy persisted despite treatment with tacrolimus ointment, clobetasol ointment, or oral fluconazole. Patient was started on a trial of baricitinib for alopecia areata, which was the suspected cause of the nail changes. After 4 months of treatment with baricitinib, the patient's nail showed mild improvement of nail dystrophy with some clubbing and pitting still present. Within 11 months of treatment, her nail was normalized in appearance and texture. There are no established guidelines to treat AA-associated nail changes. Our patient's AA-associated nail changes were normalized after 11 months of treatment with baricitinib. Further research is needed to determine which alopecia areata patients may benefit from treatment with baricitinib and when treatment should be initiated. Baricitinib may be an effective treatment option for AA-associated nail changes in some patients.

## 1. Introduction

Alopecia areata is a common, lymphocyte cell-mediated inflammatory cause of hair loss [1, 2]. During the early 1970s, a National Health and Nutrition Examination Survey study estimated the prevalence of AA in the United States to be approximately 0.1–0.2% of the population [1, 3]. Nail changes are seen in some individuals with alopecia areata, with the most common variants including pitting and trachyonychia [1, 4]. These nail changes may occur before or months to years after the hair loss from AA [1, 5]. Nail changes seen in alopecia areata are considered to be a poor prognostic factor of the condition and can be used as an indicator of the severity [2, 6].

Trachyonychia occurs secondary to lymphocytic infiltrate. Histologically, AA-associated trachyonychia is characterized by mild to moderate inflammatory infiltrate accompanied by exocytosis and spongiosis [5]. It occurs in the nail bed, matrix, folds, and hyponychium. While trachyonychia is a common nail manifestation patients with AA, its occurrence can also be due to another skin condition such as lichen planus, psoriasis, or atopic dermatitis [1, 5]. Similar to trachyonychia, pitting is due to the underlying lymphocytic infiltrate. This results in abnormal keratinization of the nail that occurs slits and longitudinal scratches of the upper edge of the nail plate [1].

The pathogenesis of AA-associated nail changes is not well understood. Currently, the nail findings are presumed to be due to the same lymphocytic infiltration seen in hair bulbs in individuals with AA [1]. When patients with AA develop nail changes, the number of nails involved varies across individuals. Some patients may have all nails affected, some nails, or just a single nail with changes [1].

There are currently limited data in the literature on therapeutic options for AA-associated nail changes [1]. There have been case reports of treating nails topically with corticosteroids, and in a single case, tazarotene 0.1% gel nightly [7, 8]. In some cases, intralesional triamcinolone has been used; however, relapses eventually occur in two-thirds of patients [7]. Systemic steroids such as oral prednisone have been for severe nail changes [7]. In 2016, Ferreira et al. described a case of nail changes in a patient with alopecia universalis that were successfully treated off-label with tofacitinib, a JAK inhibitor. Their patient's hair loss was refractory to many treatments, and nail changes included striated lunulae, pitting, trachyonychia, and onychorrhexis. The authors reported that hair growth was noted after two months of therapy and growth of all nails with normalization of the nail plates occurred after ten months [6]. A retrospective study in 2018 also showed nail improvement in a small group of patients with moderate-to-severe AA treated with tofacitinib [9].

While there are very limited data on the use of baricitinib for AA-associated nail changes, baricitinib has been used to treat cases of nail changes due to lichen planus [10]. Baricitinib is an immunomodulatory medication that acts as a selective and reversible inhibitor of JAK proteins. JAK proteins play a significant role in immune cell functioning by modulating cytokines signals and growth factor receptors. Inhibition of JAK proteins by baricitinib prevents the phosphorylation and activation of signal transducers and activators of transcription (STAT) proteins, resulting in a dampened immune response [11]. Baricitinib is indicated for adult patients with moderate to severe rheumatoid arthritis who have not responded to other disease-modifying antirheumatic drugs. Baricitinib has also been approved by the FDA to treat patients hospitalized with COVID-19, as well as severe alopecia areata. Baricitinib has also been used for severe atopic dermatitis, psoriatic arthritis, and vitiligo. While baricitinib has shown to be a successful treatment option for alopecia areata, there is minimal research to show the efficacy of baricitinib treatment on improvement of nail changes seen in patients with alopecia areata.

In this report, we present a case of a patient with nail changes due to alopecia areata who has been successfully treated with baricitinib.

## 2. Case Presentation

**Figure 1:**
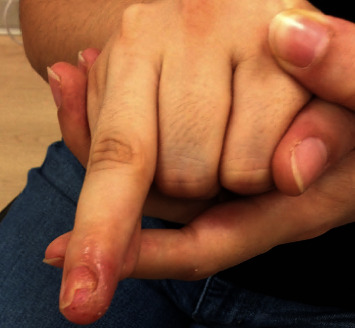
Nail changes observed at patient's first clinic visit—erythematous, inflammatory changes of the nail of the right 4th digit with pitting. No clinical changes of other nails observed.

**Figure 2:**
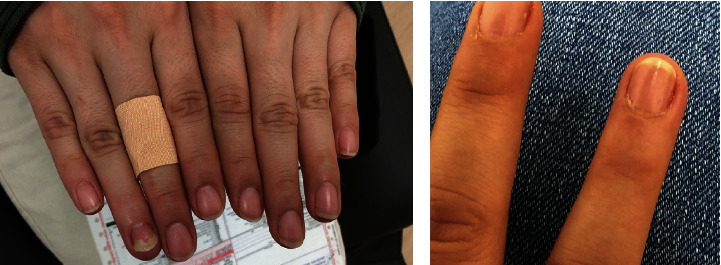
(a) Right 4th nail dystrophy with pitting on the day trial of baricitinib was initiated. (b) Right 4th nail with normalized in texture and appearance after 11 months of treatment with baricitinib.

The patient is a 28 year-old-female who presented to the clinic with alopecia areata of the scalp, eyebrows, and eyelashes. The patient has been suffering from alopecia for several years. She also presented with inflammation, persistent fingernail ridges, and pitting of her right 4th digit. The inflammation of the digit surrounding the nail improved with tacrolimus ointment, triamcinolone lotion, clobetasol ointment, and oral fluconazole over the course of eight weeks (Figure 1). However, the nail dystrophy showed no response to this treatment. Shave biopsy of the 3rd right digit performed in the clinic to rule out psoriasis revealed spongiotic dermatitis. The patient was started on a trial of baricitinib 4 mg tablets daily for alopecia areata, which was the suspected cause of the nail changes (Figure2(a)). After 4 months of treatment, the patient's nail showed mild improvement of nail dystrophy with some clubbing and pitting still present. At this time, she had experienced significant growth of her eyelashes and eyebrows. Within 11 months of treatment, her nail was normalized in appearance and texture (Figure 2(b)). Throughout her course of treatment with baricitinib, she has benefitted from overall improvement of alopecia areata-induced hair loss of her scalp, eyelashes, and eyebrows. She is currently being treated with baricitinib to this day, with no plans to discontinue therapy.

## 3. Discussion

Nail changes and disfigurement in patients with alopecia areata may potentially impact the quality of life. Dystrophic nails can make it difficult to complete daily activities with the hands and may be cosmetically concerning, leading to self-consciousness [1, 2]. AA has been shown to have a detrimental impact on the quality of life of patients, and nail changes may add to this burden [2]. There are no established guidelines to treat AA-associated nail changes. Current management options for AA-associated nail changes require a multifactorial approach. The treatment should be selected based on the type and severity of clinical signs, number of affected nails, comorbidities, previous treatments, age, and quality of life [12]. If manual function is unaffected or there are no cosmetic concerns, a wait-and-see approach may be used to see if spontaneous recovery of the nail changes occurs. There have been reports of nail changes being treated with topical, intralesional, and oral steroids; however, recurrence of the nail dystrophy often recurs [7]. Our patient's affected nail demonstrated no response to topical steroids or calcineurin inhibitors. While there has been anecdotal evidence of improvement of nail changes with tofacitinib, this drug is not currently FDA-approved to treat AA-associated nail changes [9]. Tofacitinib has been shown to improve nail related changes in patients with alopecia areata with continued remission after cessation of the drug [13]. Baricitinib is similar to tofacitinib in that it is also an oral JAK inhibitor. If nail changes have not been shown to recur after stopping treatment with tofacitinib, it is possible that they may not recur with discontinuation of baricitinib. There is limited research studying the efficacy of baricitinib on AA-associated nail changes. Studies have shown that AA relapses may occur during baricitinib treatment, which suggests the possibility of AA-associated nail related changes also relapsing during baricitinib treatment [14]. Further research is needed to determine which alopecia areata patients may benefit from treatment with baricitinib and when treatment should be initiated. Additionally, it is unclear as to whether AA-associated nail changes that resolve after treatment with baricitinib will eventually recur.

## Data Availability

Data sharing is not applicable to this article as no new data were created or analyzed in this study.
